# The Current Status and Influencing Factors of Hospitalized Patients' Propensity to Engage in Patient Safety: A Descriptive Cross-Sectional Study

**DOI:** 10.1155/jonm/2701869

**Published:** 2025-06-30

**Authors:** Ping Yuan, Cong Wang, Yan Cai, Jun Zhou, Yuanyuan Yang, Fan Zhang, Caili Li, Yan Jiang

**Affiliations:** ^1^Department of Neurosurgery, West China Hospital, Sichuan University/West China School of Nursing, Sichuan University, Chengdu 610041, Sichuan, China; ^2^Department of Nursing, The People's Hospital of Leshan, Leshan 614000, Sichuan, China; ^3^Evidence-Based Nursing Center, West China Hospital, Sichuan University, Chengdu 610041, Sichuan, China; ^4^Department of Family Medicine, The People's Hospital of Leshan, Leshan 614000, Sichuan, China; ^5^Department of Cardiovascular Medicine, The People's Hospital of Leshan, Leshan 614000, Sichuan, China; ^6^Department of Hemodialysis, The People's Hospital of Leshan, Leshan 614000, Sichuan, China; ^7^Day Surgery Center, General Practice Medical Center, West China Hospital, Sichuan University/West China School of Nursing, Sichuan University, Chengdu 610041, Sichuan, China

## Abstract

**Aims:** To investigate the current level of inpatients' propensity to engage in patient safety initiatives and pinpoint the influencing factors, thereby offering insights for targeted interventions and facilitating the development of an efficient management mechanism.

**Design:** A cross-sectional descriptive study.

**Methods:** One thousand four hundred and ninety-eight inpatients were recruited from 18 medical institutions in Leshan City, Sichuan Province, China. Participants provided their sociodemographic and professional information and completed a questionnaire scale on the willingness of Chinese inpatients to participate in patient safety. The collected data were analyzed using descriptive statistics, one-way analysis of variance, and binary logistic regression.

**Results:** The highest score for patients' propensity to participate in safety initiatives was 4.13 ± 1.19 points. Binary logistic regression analysis revealed that religion, place of residence, number of hospitalizations, occupation, and hospital grade were independent factors influencing patients' willingness to engage in patient safety (OR = 0.185, *p* < 0.001).

**Conclusion:** Patients in this region are moderately willing to participate in patient safety initiatives. However, while the survey mainly assesses willingness, the behavioral tendency subdimension reveals a gap between patients' expressed willingness and their reported engagement behaviors.

**Summary**

• Implications for the profession and/or patient care
◦ Patient engagement can improve patient safety in hospitals, thereby enhancing patient engagement and medical safety.
◦ This study identifies the current situation and influencing factors and provides a scientific basis for optimizing intervention and management strategies and strengthening patient engagement and medical safety.

## 1. Introduction

Since the Institute of Medicine (IOM) reported that inpatients are more likely to die from adverse events than from traffic accidents, patient safety has become a focal point in healthcare [1, 2]. Currently, patient safety is regarded as a critical issue in healthcare [2]. Ensuring safety is not only a fundamental right for patients but also a primary responsibility and goal for governments and healthcare professionals (HCPs) [3]. Patient safety involves delivering care without causing harm or injury to patients, with failures potentially leading to irreparable consequences. Healthcare systems worldwide face daily challenges in preventing and managing such incidents [3]. Despite extensive efforts to enhance safety, unintentional injuries caused by healthcare providers that result in serious harm to patients continue to be prevalent [4]. In the United States, medical errors are responsible for an estimated 250,000 deaths, making them the third leading cause of death [5]. Each year, approximately 134 million patients are harmed, and 2.6 million die as a result of unsafe medical practices, imposing a significant burden of death and disability globally, especially in low- and middle-income countries, further highlighting the urgent need to improve patient safety [6].

Patient engagement in safety is defined as patient-centered healthcare that encourages patients and their families to actively collaborate with HCPs in reducing or preventing harm through patient-involved behaviors [7]. Involvement in health policy, healthcare planning and improvement, and direct care is increasingly recognized as a cornerstone of quality and safety. Patient engagement in care has become a priority and a key component of clinical practice in many countries worldwide [8]. Patient-centered care strategies empower individuals to shape their treatment and care in ways that align with their needs and preferences, ultimately leading to improved outcomes [9]. Research has shown that patient participation positively influences various aspects, including patient trust in clinicians [10], patient satisfaction [11], patient adherence [12], and overall patient outcomes [13, 14]. Furthermore, patient engagement has been linked to reduced healthcare utilization [15], a decrease in adverse events [11], and the prevention of subsequent medical litigation [16].

Patient engagement is considered one of the most effective strategies to reduce medical errors, with patients' willingness to participate serving as both the prerequisite and the motivation for encouraging and guiding their involvement [8]. Therefore, understanding patients' actual willingness and needs is crucial for enhancing participation and ensuring patient safety. Although the importance of patient engagement in healthcare is increasingly recognized by the academic community, opportunities to promote genuine patient involvement remain in the early stages of development [17]. Studies have explored patients' willingness to engage in safety-related activities, revealing that while some patients are interested in participating in tasks such as medication administration and handwashing, this engagement is not universal across all wards [18]. A Swedish report highlighted that patients rated low on accessibility, information availability, and engagement opportunities, with not all wards actively promoting patient participation [17]. While these studies provide valuable insights, they have notable limitations, such as small sample sizes, data originating from single healthcare institutions, and insufficient exploration of factors influencing patients' willingness to engage. Furthermore, they lack a thorough discussion of individual patient differences (e.g., religious beliefs, occupational backgrounds, and frequency of hospitalization) and hospital-level disparities. As a result, the proposed improvements and management mechanisms have limited applicability. This study, therefore, aims to assess the current state of patients' willingness to participate in different types of healthcare organizations and identify the factors that influence their engagement. The findings are expected to provide a foundation for developing targeted interventions and establishing effective management strategies.

### 1.1. Theory of Planned Behavior (TPB) Framework

The TPB, proposed by Ajzen in 1985, is a widely validated theoretical framework for explaining and predicting human behavior [19]. The theory posits three fundamental antecedents of behavioral intention: (1) attitude toward the behavior (an individual's positive or negative evaluation of performing the target behavior), (2) subjective norm (perceived social pressure to engage or not engage in the behavior), and (3) perceived behavioral control (PBC; the perceived ease or difficulty of performing the behavior) [20]. These constructs originate from corresponding belief systems: behavioral beliefs form attitudes, normative beliefs shape subjective norms, and control beliefs constitute PBC [21]. Together, these factors predict behavioral intention, which, along with PBC, directly influences actual behavior.

This study employs the TPB framework to investigate hospitalized patients' willingness to participate in patient safety (Patient Participates Patient Safety Willingness and Behavior Scale, PSWBS) initiatives, offering three key theoretical contributions:

1. Needs' assessment: Measuring behavioral attitudes reveals patients' cognitive-affective tendencies toward safety engagement
2. Belief reinforcement: Analyzing subjective norms identifies social influence factors in clinician–patient interactions
3. Capability building: Evaluating PBC pinpoints' behavioral barriers to patient participation

Compared to traditional behavioral theories, TPB's unique strength lies in its simultaneous consideration of internal motivation (attitude) and external constraints (control) [21], making it particularly suitable for examining patient decision-making in the power-asymmetric healthcare environment. This theoretical perspective provides essential analytical tools for constructing the “intention-behavior” pathway in our study.

## 2. Methods

### 2.1. Design and Setting

This cross-sectional study was conducted in Leshan City, Sichuan Province, China, from December 2020 to January 2021. A stratified cluster random sampling method was employed, with stratification based on hospital level. The proportion of inpatients at the 18 different levels of medical institutions—tertiary Class A, tertiary Class B, secondary Class A, and secondary Class B—was used to determine the sample size for each hospital level. Using the random number table method, 50% of the hospitals at each level were randomly selected as study subjects. This article adheres to the STROBE guidelines.

#### 2.1.1. Participants

Participants in this study were inpatients. The sample size was calculated using G-Power 3.1.9.7 software. Considering logistic regression, *⍺* = 0.05, 1 − *β* = 0.80, odds ratio = 1.3, and a 20% attrition rate, at least 710 patients were needed. In this study, an electronic questionnaire was created for data collection through the Questionnaire Star platform. Before the formal survey, first, the researcher chose to select a presurvey with a sample size of 100 hospitalized patients at one time in different tiers of healthcare in the region to ensure the clarity of the questionnaire. The researcher then explained the purpose and importance of the study and the method of data collection to each hospital nursing department. The purpose and significance of the study were explained to the study participants before the survey. The same IP address could only be filled out once. The survey data were kept confidential to protect the privacy of the participants. A total of 1560 questionnaires were distributed in this study, including 468 questionnaires in Tertiary A hospitals, 187 questionnaires in Tertiary B hospitals, 746 questionnaires in Secondary A hospitals, and 159 questionnaires in Secondary B hospitals; 1560 questionnaires were retrieved, with a recovery rate of 100%, of which 62 were excluded due to a completion time of less than 60 s or incomplete answers. Finally, 1498 questionnaires were analyzed.

#### 2.1.2. Inclusion and Exclusion Criteria

Inclusion criteria include the following: (1) patients aged ≥ 18 years, (2) patients who are hospitalized for 3 days or more in medicine or surgery and discharged within 24 h, (3) no medical conflicts during hospitalization, with full cognitive and behavioral capacity, and (4) informed consent and voluntary participants. Exclusion criteria include the following: those participating in other similar clinical trials and those who completed the questionnaire incorrectly or missed items. To reduce bias caused by differences in regional medical standards and to ensure the comparability of research data, this study classifies participants into two major groups—medical and surgical—based on the principle outlined in Surgery [22], which states that “surgical diseases are primarily treated through surgical procedures.” Specifically, the medical group includes patients who received conservative treatment (e.g., medication or clinical observation), while the surgical group includes patients who required surgical intervention (including both emergency and elective surgeries).

#### 2.1.3. Instruments

Two measures were used in this study. The first was a self-developed questionnaire collecting sociodemographic and professional information. The second was an established scale assessing patient safety engagement across multiple dimensions.

#### 2.1.4. Self-Designed Sociodemographic and Professional Information

The measure consisted of 15 questions and focused on the sociodemographic characteristics of the participants, including gender, age, marital status, religious beliefs, ethnicity, educational level, place of residence, health insurance, occupation, monthly household income, inpatient department, length of stay, type of admission, type of hospital, and hospital level.

#### 2.1.5. The PSWBS

Guided by the PSCHO theoretical paradigm [23], the research team led by Chinese scholar Li et al. [24] conceptualized the PSWBS as comprising four distinct dimensions: willingness tendency, processing technology tendency, environmental safety tendency, and behavior tendency. This 14-item instrument demonstrated satisfactory psychometric properties, with Cronbach's α coefficient of 0.87 indicating strong internal consistency and measurement validity. Employing a graded response format (5 = *very concerned*, 4 = *concerned*, 3 = *not certain*, 2 = *not concerned*, 1 = *very unconcerned*). The higher the score, the greater the willingness to engage in patient safety, and the more pronounced this behavioral tendency becomes.

### 2.2. Data Analysis

Statistical analysis was performed with SPSS 25.0. Categorical data are described as frequencies and percentages (%), and quantitative data conforming to normal distribution are described as means ± standard deviations (*x* ± *s*). Binary logistic regression analysis was used to analyze the influencing factors. The mean willingness score was used as the cutoff to divide the group into high and low groups, and a multiple-factor analysis was performed using the willingness score as the dependent variable. Age, marital status, religious belief, education level, place of residence, type of medical insurance, number of hospitalizations, type of admission, average monthly household income, occupation, type of hospital, and level of hospital were included as independent variables in the regression equation for the analysis. A difference was considered statistically significant *p* < 0.05.

### 2.3. Ethical Considerations

This study was conducted after approval by the institutional review board of the corresponding author's institution (Approval no. 2021005). All participants were allowed to read the details of the study and decide whether to voluntarily participate.

## 3. Results

### 3.1. General Characteristics of Participants

Table 1 shows the demographic and professional characteristics of the participants. The final sample consisted of 1498 participants: 448 (29.9%) from Tertiary A medical institutions, 176 (11.7%) from Tertiary B medical institutions, 717 (47.9%) from Secondary A medical institutions, 157 (10.5%) from Secondary B medical institutions, and 1187 (79.2%) from outpatient clinics. Most were female (*n* = 927, 61.9%), married (*n* = 1161, 77.5%), and had a college degree or higher (*n* = 429, 28.6%), most lived in a rural area (*n* = 696, 46.5%), had been hospitalized once (*n* = 807, 53.9%), and were from a general hospital facility (*n* = 1037, 69.2%).

### 3.2. Total Willingness Score and Scores for Each Dimension

The survey results showed that the overall score for patients' willingness to participate in patient safety was (53.01 ± 14.44) points. The mean scores for the dimensions, from high to low, were as follows: willingness dimension (4.13 ± 1.19) points, tendency to pay attention to diagnosis and treatment techniques (3.86 ± 1.32) points, tendency to pay attention to environmental safety (3.74 ± 1.30) points, and tendency to participate in behavior (3.07 ± 1.25) points. The highest score was for willingness, and the lowest score was for the tendency to participate in behavior (Table 2).

The scores for each item showed that the highest scores were for “willingness to ask doctors and nurses about their condition and related circumstances” and “willingness to ask doctors about the entire treatment plan;” the lowest scores were for “taking the initiative to ask nurses whether the oral medication or infusion is correct when they come to administer it” and “taking the initiative to ask nurses whether the oral medication or infusion is correct when they come to administer it” (Table 3).

### 3.3. Analysis of Factors Influencing Willingness

The regression analysis yielded the following results: religious belief, place of residence, number of hospitalizations, occupation, and hospital level were identified as independent influencing factors of patients' willingness to participate in patient safety. The regression analysis revealed that religious belief was positively associated with willingness to participate in patient safety (OR = 1.817, 95% CI 1.085–3.045, *p*=0.023), while negative associations were observed for rural residence (OR = 0.507, 95% CI 0.370–0.694, *p* < 0.001), 3–5 hospital admissions (OR = 0.660, 95% CI 0.472–0.921, *p*=0.015), and student/unemployed/other occupational status (OR = 0.707, 95% CI 0.534–0.937, *p*=0.016). These findings suggest that while religious affiliation may enhance engagement, certain demographic and socioeconomic factors may serve as barriers to patient participation in safety initiatives. Additionally, patients treated at Tertiary B and Secondary A hospitals demonstrated a negative correlation with patients' willingness to participate in patient safety (OR 0.364, 95% CI 0.250–0.528, *p* < 0.001; OR 0.509, 95% CI 0.390–0.664, *p* < 0.001) and gender, age, marital status, ethnicity, education level, type of medical insurance, department of hospitalization, mode of admission, average monthly family income, type of hospital, nature of hospital, and patient's willingness to participate in patient safety were not statistically significant (*p* > 0.05), as shown in Table 4.

## 4. Discussion

This survey, conducted among 1498 patients across 18 healthcare organizations, revealed three key findings. First, the propensity of hospitalized patients to engage in safety initiatives was at a moderate level. Second, patients' willingness to participate was predominantly passive, with fewer patients taking concrete actions. Finally, religious affiliation, place of residence, number of hospitalizations, occupation, and hospital class were identified as predictors of inpatients' propensity to engage in safety-related activities. These findings highlight the need for practical strategies to raise awareness of inpatient involvement in safety and to encourage more active participation in managing their safety during hospitalization.

The results of this study indicated that the overall score for the willingness to participate in patient safety among hospitalized patients in the region was 53.01 ± 14.44, which is consistent with the findings of Singer et al. [25], but significantly lower than the results reported by Ashinyo et al. [26]. These findings suggest that hospitalized patients exhibit a relatively low level of willingness to engage in safety-related activities. Dimensional analyses revealed that the highest scores were in the willingness dimension, while the lowest scores were in the actual tendency to engage in behaviors. This indicates that patients' engagement in safety is primarily at the cognitive level, with limited action taken to implement safety measures. Previous studies have similarly reported this finding [26]. The mean scores of the individual items showed that “actively communicating with doctors and nurses and asking for knowledge about treatment” ranked third-lowest among all items, suggesting that patient participation remains predominantly passive. Despite patients' strong willingness, they do not take sufficient action in practice. This may be attributed to factors such as lack of knowledge, low self-confidence, fear of doctor–patient conflict, cultural and social influences, and a lack of trust in HCPs [27]. Moreover, the lowest-scoring item was “patient-initiated inquiry about correct medication when the nurse dispenses oral medication or infusion,” further highlighting that the sharing of safety information between physicians and patients is selective, with some important details potentially not communicated adequately. These findings suggest that hospitals and departments have not achieved the desired outcomes in implementing their strategic plans, underscoring the urgent need for initiatives to enhance patient engagement. However, improving patient engagement in safety management is a complex task that requires a multifaceted approach, including enhancing patient education, creating opportunities for active involvement, and fostering a positive environment for physician–patient communication.

Individual patient characteristics demonstrated significant associations with safety engagement, with religious beliefs showing a particularly notable positive influence. The religiosity effect likely operates through several interconnected pathways. First, religious values often emphasize altruism and collective responsibility, creating natural alignment with patient safety goals, as observed in Johnstone et al.'s [28] cross-cultural studies. Second, evidence from Chinese clinical setting [29] suggests religious patients may develop stronger trust in healthcare providers, facilitating more active participation. Additionally, the coping resources provided by spiritual beliefs can help reduce hospitalization-related anxiety, thereby freeing cognitive capacity for safety-related engagement. These mechanisms collectively suggest that culturally attuned interventions could strategically frame safety participation as both a moral duty and community benefit, potentially enhancing engagement among religious populations while respecting diverse value systems.

The place of residence also significantly affects patients' willingness to participate. Urban patients tend to exhibit a higher willingness to engage due to better healthcare resources, facilities, and health education support. In contrast, patients in townships and rural areas display relatively lower levels of participation, owing to limited healthcare resources, insufficient health education, and concerns over financial burdens [30–32]. The economic and educational disparities between urban and rural areas further contribute to the differences in patient engagement. Botchwey et al. [32] showed that township residents have lower literacy and income levels than their urban counterparts, which may diminish their awareness and willingness to respond to the need for healthcare coverage. Leshan is a region in southwestern China with a mixed urban–rural structure, a predominantly aging population, and relatively underdeveloped healthcare infrastructure in its rural counties and towns. Previous studies have shown that regional disparities in socioeconomic status and health literacy can significantly affect patient safety engagement [33]. Therefore, to reduce the urban–rural gap, future care policies and management should focus more on safety education and patient engagement awareness in primary care. The number of hospitalizations likewise had a differential effect on patients' willingness to participate in safety. In our study, patients from rural areas or lower-tier hospitals exhibited lower willingness to engage in safety, likely due to reduced access to health education, fewer interactive opportunities with healthcare providers, and limited patient empowerment mechanisms. These regional characteristics support the interpretation that contextual socioeconomic and healthcare system variables are critical to understand variations in safety participation.

The study revealed an inverse relationship between hospitalization frequency and willingness to engage in safety behaviors. Patients with 3–5 hospitalizations exhibited lower participation motivation, potentially due to growing dependency on healthcare systems. In contrast, patients with more than five hospitalizations—who were likely exposed to complex care scenarios or safety incidents—demonstrated entrenched reliance on clinicians, possibly because they perceived their own safety participation efforts as burdensome. This aligns with Segura-García et al.'s study [34], where lived experiences shaped risk perception. Similar patterns have been observed in high-income countries, though with notable differences. For example, a US-based study by Hibbard et al. found that frequent hospitalization (≥ 3 admissions/year) was associated with higher patient activation in safety behaviors, attributed to structured patient education programs in US hospitals [35]. Conversely, Davis et al. reported that patients with recurrent hospitalizations in the National Health Service (NHS) were more likely to disengage from safety behaviors due to perceived inefficacy of individual actions within a system-driven model. These contrasts highlight the mediating role of healthcare systems and cultural norms in patient participation. Targeted interventions are thus needed for frequently hospitalized patients, especially the 3–5 hospitalization cohort, to mitigate dependency [36].

Previous literature supports the notion that hospitalization experience can shape patients' attitudes and behaviors regarding safety. For example, Segura-García et al. [34] found that patients with more prior hospitalizations often exhibit adaptive coping mechanisms or learned reliance on healthcare providers, which may reduce their motivation to engage actively. Similarly, Berger et al. observed that patients' willingness to participate in safety activities varied with their prior exposure to adverse events and level of trust in the care team [37]. However, studies specifically examining how hospitalization frequency influences safety engagement in Chinese populations remain limited, indicating a need for further exploration. Therefore, targeted interventions are needed for frequently hospitalized patients, especially those in the 3–5 admission cohort, to mitigate dependency. Clinicians should reinforce safety education, emphasizing collaborative roles even among high-admission patients to sustain proactive engagement.

Occupational factors are another crucial variables influencing patient safety participation. These factors can enhance awareness of safety issues and encourage more individuals to actively engage in safety management [38]. Work experience, tenure, and personal confidence all affect individuals' perceptions, judgments, and decisions [39]. HCPs' understanding of safety practices tends to improve with experience [39]. Additionally, there are notable differences in safety culture performance across various occupational fields [40, 41], which aligns with our previous findings [42, 43].

We also observed a lower willingness to participate among unemployed individuals and students, which is consistent with the findings of Huang et al. [44]. This may be attributed to their pressing financial concerns that prioritize immediate healthcare costs over safety participation, coupled with lack of experience in healthcare and limited safety awareness. Additionally, this group, due to their relatively flexible lifestyles, greater leisure time, and better compliance during hospitalization, often tends to delegate medical responsibilities entirely to HCPs, neglecting their role in safety management. As a result, this group is more likely to rely on HCPs for decision-making and lacks the intrinsic motivation to participate actively in their care. However, the interactive behaviors of HCPs can encourage patient involvement in the treatment process [45]. Therefore, hospitals should enhance safety education for students and unemployed patients by providing personalized education and psychological support to increase their awareness and active participation, ultimately improving the effectiveness of safety management.

This study also revealed a significant correlation between hospital class and the propensity of hospitalized patients to participate. Davis et al. [46] similarly demonstrated that hospital class, healthcare setting, and level of care were strongly associated with patients' willingness to engage in their care. This relationship is closely linked to differences in resource allocation and service capacity across healthcare organizations. Patients in tertiary care hospitals were found to have a higher propensity to participate, likely due to several factors. First, as regional medical centers, tertiary hospitals typically have abundant resources and higher medical service capacity, which not only enhances patients' trust in the quality of care but also provides more opportunities for them to participate in medical decision-making [46]. Additionally, these hospitals often feature well-developed health education systems, which can more effectively promote patients' safety awareness and participation in their care [47].

Second, tertiary hospitals tend to have more transparent and open communication between patients and healthcare providers, as well as a more established safety culture, which fosters an environment conducive to patient involvement. In contrast, limitations in medical conditions and insufficient information exchange in lower-level hospitals may lead to a lack of patient awareness about their role in healthcare, which is reflected in a reduced willingness to participate [47]. Therefore, the influence of hospital class on patient safety management should not be overlooked. Future interventions should be tailored to the characteristics of different healthcare settings. For tertiary hospitals, personalized participation strategies should be optimized, and efforts to strengthen the patient safety culture should be prioritized. In contrast, for lower-tier hospitals, the focus should be on enhancing patient education, improving information transparency, cultivating safety awareness, and reducing disparities in participation willingness due to differences in healthcare resources.

### 4.1. Implications for Policy and Practice

The results of this study revealed the propensity and factors influencing hospitalized patients' willingness to engage in patient safety, with important policy, practice, and care management implications. First, at the policy level, governments and health authorities should promote more targeted patient safety management programs and implement the key objectives of the Global Action Plan for Patient Safety 2021–2030, especially patient and family engagement as an important strategy to achieve “zero harm”. Given the positive impact of religious beliefs on patients' willingness to participate, policies could consider strengthening healthcare safety education through community and religious institutions, especially for patients with religious beliefs, and utilizing the positive power of their beliefs to enhance their motivation to participate in healthcare safety management. In addition, to address urban–rural differences, the government should promote a balanced allocation of urban and rural medical resources through policies that increase safety education and facility support for township- and village-level medical institutions to narrow the gap between urban and rural patients in terms of their participation in safety management. At the practice level, medical institutions should develop personalized participation strategies for different patient groups. For patients who have been hospitalized multiple times, their awareness of participation should be strengthened to avoid dependency and help them participate more actively in safety management. HCPs should provide students and unemployed patients with more detailed safety education and psychological support to enhance their willingness to participate. In addition, hospitals should encourage more open and transparent doctor–patient communication to reduce patients' doubts about medical safety and stimulate their motivation to participate.

### 4.2. Strengths and Limitations of the Work

This study has several strengths. First, it is one of the few studies describing the propensity of hospitalized patients' willingness to participate in patient safety at different levels of care. This study investigated an adequate sample of patients in different levels of hospitals and was able to provide a more comprehensive picture of the current status of patient's willingness to participate in various types of healthcare organizations. Second, based on inpatient data from the Leshan region, this study makes up for the lack of research on patient safety participation in this region in previous studies, which is representative and informative, and for the first time delves into the influence of factors such as sociodemographic characteristics and the grade of healthcare organization on the propensity of patients' willingness to participate in patient safety. The findings of this study provide a basis for HCPs to gain an in-depth understanding of the basics of patient safety engagement, as well as a reference for healthcare organizations, to develop targeted interventions to help better promote patient engagement in safety management. However, there are some limitations to this study. First, the study only conducted a cross-sectional survey and lacked implementation and evaluation of interventions. Second, the study sample was only from secondary and higher-level healthcare organizations in the region and did not cover primary healthcare organizations and township health centers, which has certain regional limitations and limited extrapolation of the results.

## 5. Conclusion

In conclusion, the willingness to participate in patient safety among hospitalized patients in secondary and higher medical institutions in Leshan City is high and at a medium level. However, a gap was identified between patients' reported willingness and their self-reported behavioral tendency to engage in patient safety activities. This discrepancy suggests a potential intention–action divide rather than a measure of objectively observed behavior. Furthermore, factors such as religious belief, place of residence, number of hospitalizations, occupation, and hospital level were found to significantly influence patients' willingness to participate. Patients' willingness to participate in safety management mostly remained at the ideological level with limited concrete behavior, which reflects their lack of knowledge, confidence, and doctor–patient communication. Therefore, when developing future programs to enhance patient safety management, it is recommended to consider the needs and characteristics of different groups fully, paying special attention to rural patients, frequently hospitalized patients, and patients from lower occupational classes, and providing targeted education and early intervention. At the same time, patient participation can be further enhanced by strengthening information transparency and trust between doctors and patients, and establishing more open communication channels, thus better protecting patient safety.

## Figures and Tables

**Table 1:** General characteristics of the participants.

Characteristics	Categories	Total (*N* = 1498) *n* (%)
Gender	Male	571 (38.1)
	Female	927 (61.9)

Age, year	< 39 years	521 (34.9)
	40–59 years	538 (35.9)
	≥ 60 years	439 (29.3)

Marital status	Unmarried	196 (13.1)
	Married	1161 (77.5)
	Widowed/divorced	141 (9.4)

Education level	Primary school and below	500 (33.4)
	Junior high school–technical high school	569 (38.0)
	College and above	429 (28.6)

Residence	City	380 (25.4)
	County city	422 (28.2)
	Village	696 (46.5)

Medical insurance	Health insurance for employees	503 (33.6)
	Medical insurance for urban and rural residents	895 (59.7)
	Others	100 (6.7)

Average household income per month	Less than 1000 yuan	289 (19.3)
	1000–3000 yuan	520 (34.7)
	3000–5000 yuan	462 (30.8)
	5000–10,000 yuan	170 (11.3)
	More than 10,000 yuan	57 (3.8)

Occupation	Farmer/worker	867 (57.9)
	Administrative/corporate employee	268 (17.9)
	Student/unemployed/others	363 (24.2)

Number of hospitalizations	1 time	807 (53.9)
	2 times	330 (22.0)
	3–5 times	208 (13.9)
	> 5 times	153 (10.2)

Hospital department	Surgery	713 (47.6)
	Internal Medicine	785 (52.4)

Admission method	Outpatient	1187 (79.2)
	Emergency/hospital transfer	311 (20.8)

Hospital categories	General hospital	1037 (69.2)
	Traditional Chinese medicine/specialty hospital	461 (30.8)

Nature of hospitals	Public hospital	1295 (86.4)
	Private hospital	203 (13.6)

Hospital grade	Tertiary Class A	448 (29.9)
	Tertiary Class B	176 (11.7)
	Secondary Class A	717 (47.9)
	Secondary Class B	157 (10.5)

Religious affiliation	None	1423 (95.0)
	Buddhism	35 (2.3)
	Christianity	6 (0.4)
	Others	34 (2.3)

**Table 2:** Total score and scores for each dimension of patient engagement in patient safety.

Dimension	Score (mean ± SD)	Dimension mean score (mean ± SD)
Willingness tendency	24.82 ± 7.19	4.13 ± 1.19
Processing technology tendency	7.32 ± 2.66	3.86 ± 1.32
Environmental safety tendency	11.23 ± 3.92	3.74 ± 1.30
Behavioral tendency	9.24 ± 3.75	3.07 ± 1.25
Total score	53.01 ± 14.44	3.70 ± 1.25

**Table 3:** Patient participation patient safety willingness tendency score of each item.

Items	Score (mean ± SD)
1. Be willing to ask doctors and nurses about your condition and related circumstances.	4.23 ± 1.30
2. Be willing to ask doctors about the entire treatment plan.	4.20 ± 1.32
3. Be willing to ask doctors and nurses about the therapeutic effects and side effects of the oral and infusion medications they are taking.	4.10 ± 1.35
4. Be willing to ask doctors and nurses about the reasons for and the results of any tests they need to undergo.	4.10 ± 1.35
5. If undergoing surgery or treatment, be prepared to ask doctors and nurses detailed questions about the procedure and risks of the surgery or treatment.	4.09 ± 1.36
6. Before surgery or treatment, I am willing to understand the contents of the signed consent form for surgery or treatment.	4.07 ± 1.37
7. I will pay attention to the technical level of the doctors or nurses in charge of me.	3.86 ± 1.42
8. I pay attention to the service attitude of the attending doctors or nurses.	3.85 ± 1.42
9. I pay attention to whether the doctor or nurse washes his/her hands before and after treating me.	3.67 ± 1.41
10. I pay attention to whether the medical equipment and bed linen used for my operation or treatment are safe and disinfected.	3.80 ± 1.42
11. I look at whether the hospital environment, equipment, fire safety facilities, etc. are safe.	3.75 ± 1.43
12. Take the initiative to communicate with doctors and nurses, describing your condition and asking for knowledge related to your treatment.	3.20 ± 1.37
13. When the nurse comes to give oral medication or an infusion, take the initiative to ask the nurse if the medication is correct.	3.10 ± 1.37
14. Take the initiative to express your concerns and doubts about the technical level of doctors and nurses to doctors and nurses.	2.92 ± 1.45

**Table 4:** Factors influencing patients' willingness to participate in safety.

Characteristics	Categories	*B*	SE	Wald	df	*p*	OR (95% CI)
Gender	Male						1
	Female	0.030	0.118	0.063	1	0.801	1.030 (0.818–1.297)

Age, year	< 39 years						1
	40–59 years	−0.164	0.164	1.007	1	0.316	0.848 (0.615–1.170)
	≥ 60 years	−0.347	0.199	3.038	1	0.081	0.706 (0.478–1.044)

Marital status	Unmarried						1
	Married	−0.064	0.19	0.114	1	0.735	0.938 (0.647–1.361)
	Widowed/divorced	−0.217	0.269	0.651	1	0.420	0.805 (0.475–1.364)

Religious	No						1
	Yes	0.597	0.263	5.146	1	0.023	1.817 (1.085–3.045)

Ethnic	Han Chinese						1
	Ethnic minority	−0.230	0.291	0.626	1	0.429	1.258 (0.712–2.224)

Education level	Primary school and below						1
	Junior high school–technical high school	−0.027	0.145	0.034	1	0.853	1.027 (0.774–1.364)
	College and above	0.110	0.218	0.256	1	0.613	0.895 (0.584–1.373)

Residence	City						1
	County city	−0.242	0.156	2.410	1	0.121	0.785 (0.579–1.065)
	Village	−0.680	0.161	17.894	1	< 0.001	0.507 (0.370–0.694)

Medical insurance	Health insurance for employees						1
	Medical insurance for urban and rural residents	−0.133	0.151	0.776	1	0.947	1.022 (0.530–1.973)
	Others	−0.035	0.251	0.020	1	0.888	1.036 (0.633–1.694)

Number of hospitalizations	1 time						1
	2 times	−0.056	0.14	0.159	1	0.690	0.946 (0.719–1.244)
	3–5 times	−0.416	0.17	5.973	1	0.015	0.660 (0.472–0.921)
	> 5 times	−0.075	0.2	0.140	1	0.708	0.928 (0.627–1.373)

Admission method	Outpatient						1
	Emergency/hospital transfer	0.007	0.138	0.003	1	0.959	1.007 (0.768–1.321)

Average household income per month	Less than 1000 yuan						1
	1000–3000 yuan	−0.100	0.159	0.398	1	0.528	0.905 (0.663–1.235)
	3000–5000 yuan	−0.314	0.174	3.275	1	0.070	0.730 (0.520–1.026)
	5000–10,000 yuan	−0.222	0.227	0.953	1	0.329	0.801 (0.514–1.250)
	More than 10,000 yuan	−0.118	0.318	0.138	1	0.710	0.889 (0.476–1.657)

Occupation	Farmer/worker						1
	Administrative/corporate employee	−0.093	0.197	0.223	1	0.637	0.911 (0.619–1.341)
	Student/unemployed/others	−0.346	0.143	5.833	1	0.016	0.707 (0.534–0.937)

Hospital categories	General hospital						1
	Traditional Chinese medicine/specialty hospital	0.056	0.122	0.208	1	0.648	1.057 (0.833–1.342)

Nature of hospitals	Public hospital						1
	Private hospital	−0.099	0.179	0.307	1	0.579	0.906 (0.638–1.285)

Hospital grade	Tertiary Class A						1
	Tertiary Class B	−1.012	0.190	28.264	1	< 0.001	0.364 (0.250–0.528)
	Secondary Class A	−0.676	0.136	24.790	1	< 0.001	0.509 (0.390–0.664)
	Secondary Class B	−0.352	0.219	2.600	1	0.107	0.703 (0.458–1.079)

## Data Availability

The data that support the findings of this study are available from the corresponding author upon reasonable request.
